# Supplementary material for: A retinoid analogue, TTNPB, promotes clonal expansion of human pluripotent stem cells by upregulating CLDN2 and HoxA1
Source: Commun Biol. 2024 Feb 16;7:190. doi: 10.1038/s42003-024-05812-7 (PMC10873380; doi:10.1038/s42003-024-05812-7)
Supplement: Supplementary file 2 — Supplementary Figs. [file 42003_2024_5812_MOESM2_ESM.pdf]

**A Retinoid Analogue, TTNPB, Promotes Clonal Expansion of Human Pluripotent Stem Cells by Upregulating *CLDN2* and *HoxA1***

Suman C. Nath[1, 2], Shahnaz Babaei-Abraki[1], Guoliang Meng[1], Kali A. Heale[1], Charlie Y. M. Hsu[1], Derrick E. Rancourt[1, 2*]

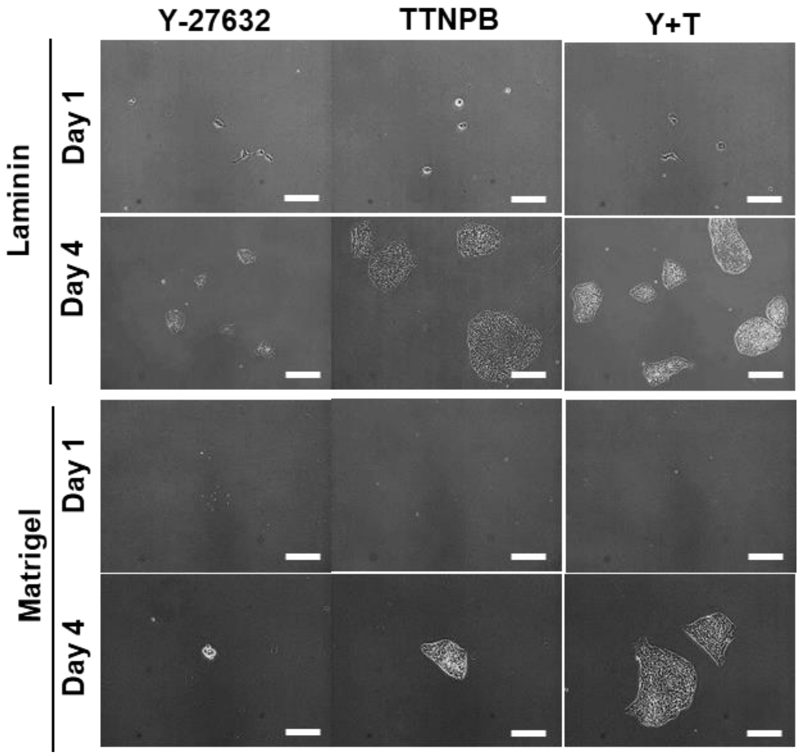


**Supplementary Figure 1**. Comparison of clonal colony formation on plates coated with Laminin and Matrigel. Brightfield images of clonal colonies on day 1 and day 4 on Laminin and Matrigel coated plates. Scale bars: 100 µm.

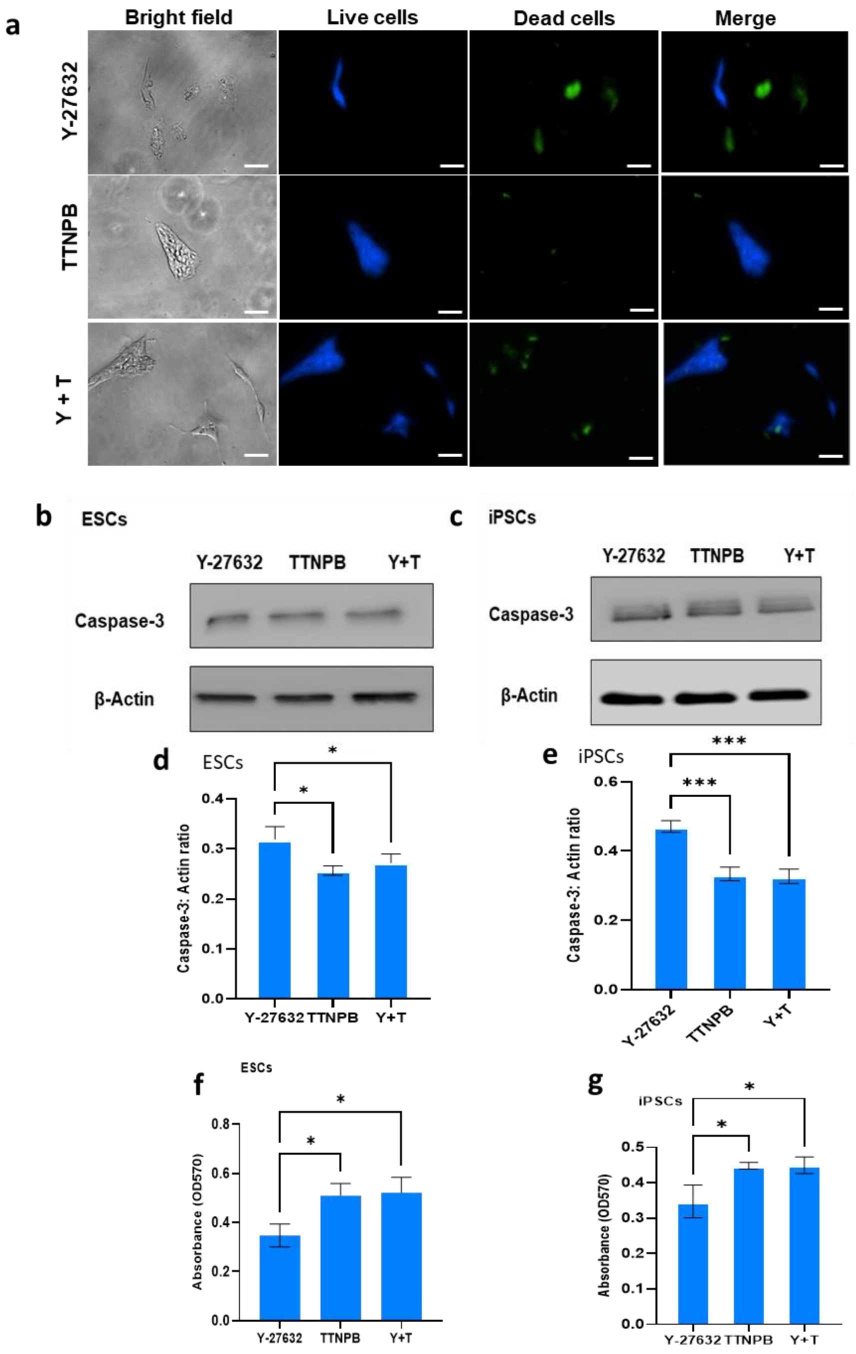


**Supplementary Figure 2**. (**a**) Confocal images of live/dead assay of hESCs 24h after treatment with Y-27632, TTNPB and Y-27632+TTNPB. Blue color indicates live, and green indicates dead cells. (**b, c**) Caspase-3 protein expression was measured using Western blot. (**d, e**) Densitometric analysis of Caspase-3 protein levels. (**f, g**) Cell adhesion of dissociated single cells cultured with Y-27632, TTNPB and Y-27632+TTNPB 6h after seeding. Data represented from N=3 experiments. *$p$ < 0.05. ***$p$ < 0.0001. Scale bars: 50 µm.

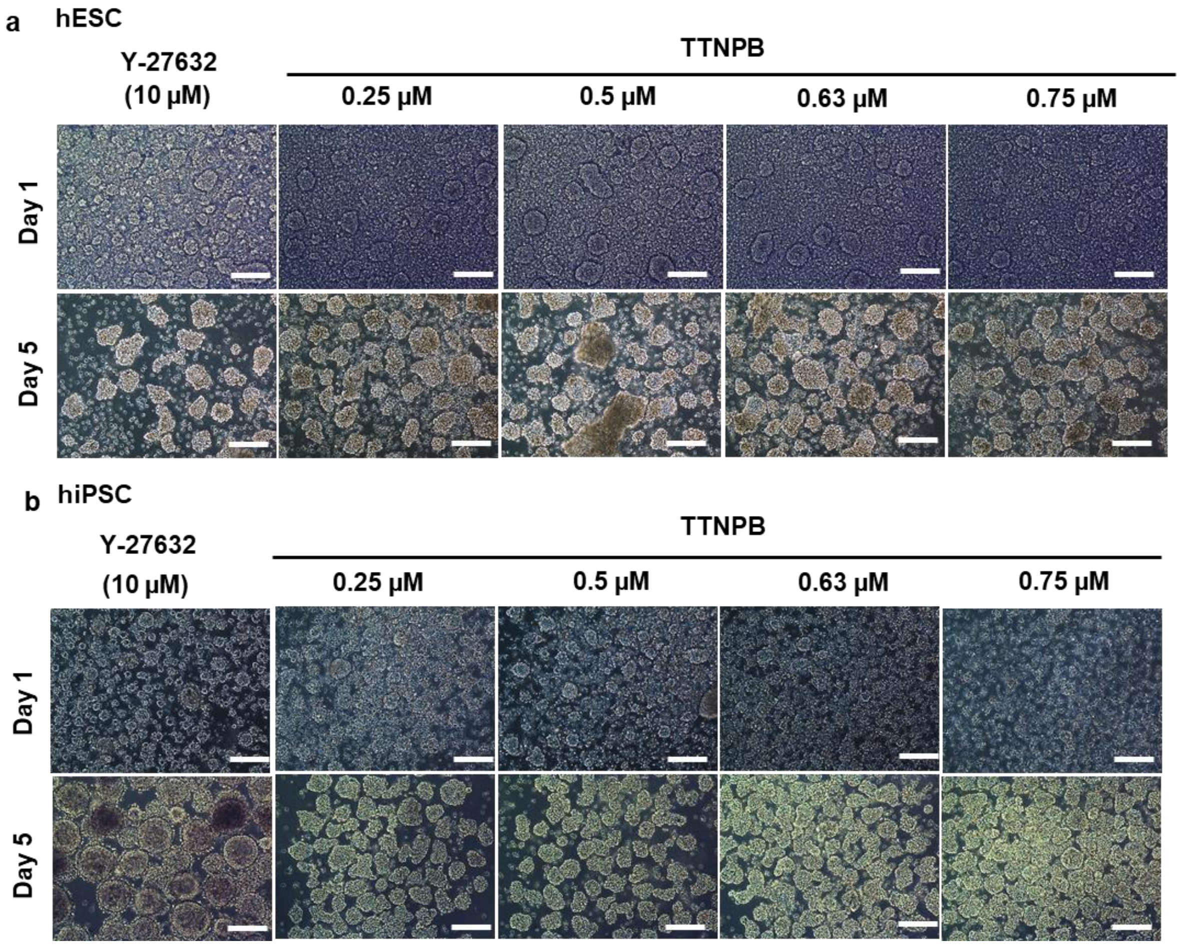


**Supplementary Figure 3**. Screening of TTNPB concentration for better aggregate formation. Bright field images of aggregates after treatment with four different concentrations of TTNPB for (**a**) ESCs and (**b**) iPSCs on day 1 and day 5. Scale bars: 100 μm.

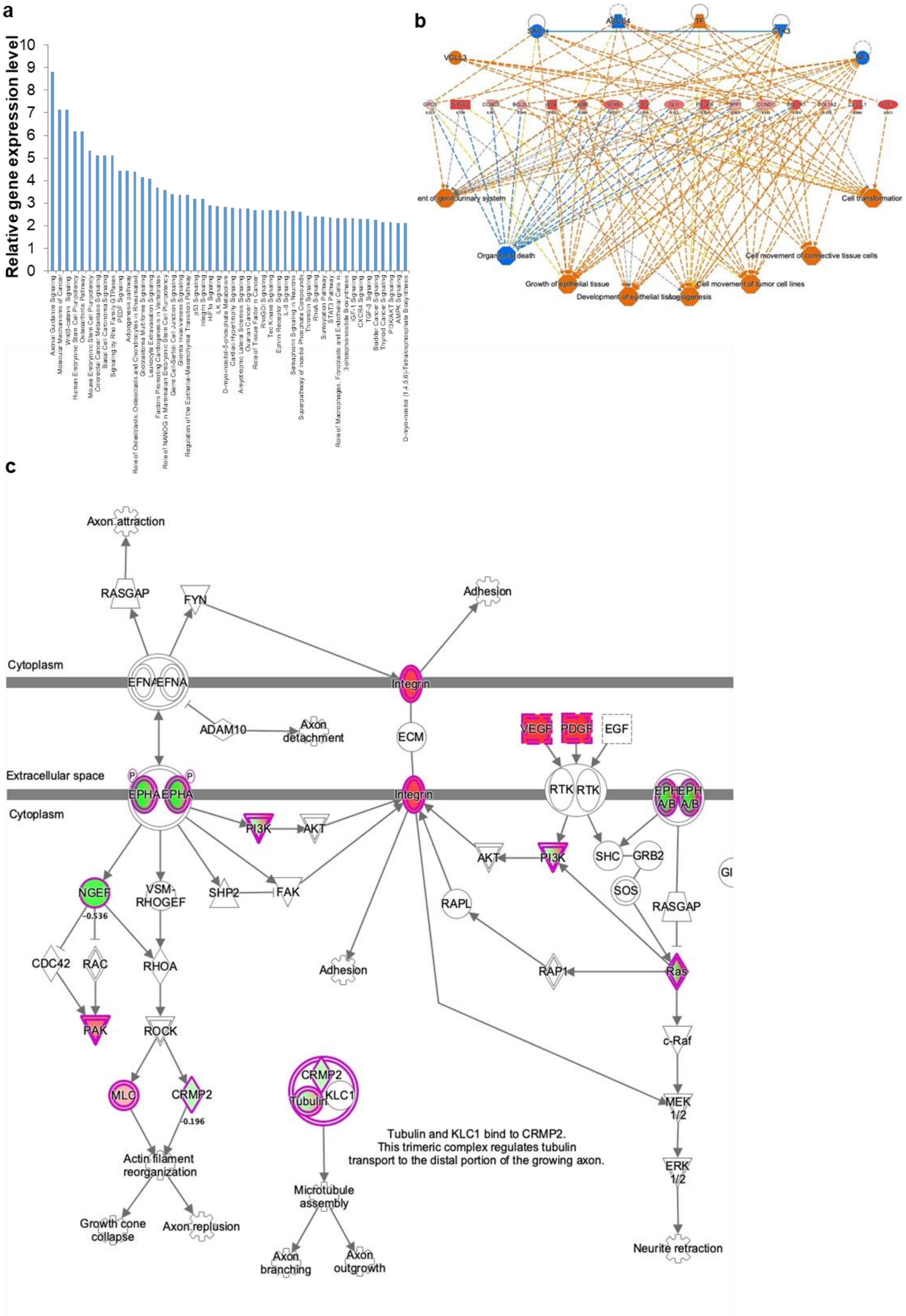


**Supplementary Figure 4**. RNA-seq analysis data of TTNPB-treated hPSCs. (**a**) Relative gene expression of canonical pathways involved with TTNPB exposure. (**b**) Cellular network of genes and their related pathways for growth and development. (**c**) Schematic of cell-cell connection and associated cell signaling genes in TTNPB-treated cells.

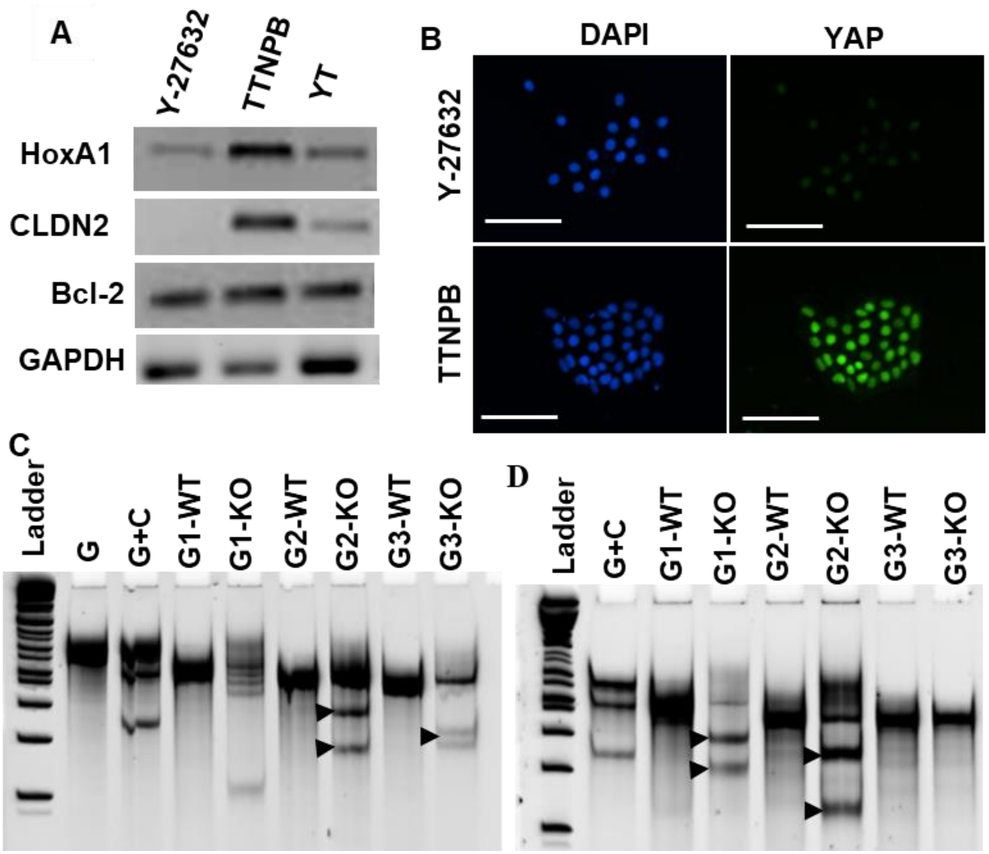


**Supplementary Figure 5**. Expression of *CLDN2* and *HoxA1* genes in TTNPB-treated hPSCs and generating their knockout clones. (**a**) *CLDN2* and *HoxA1* gene expression detected by RT-PCR in Y-27632 and TTNPB-treated cells. (**b**) Immunofluorescent staining of YAP in hESCs treated with Y-27632 and TTNPB after 48 hours of plate culture. (**c**) PCR surveyor assay for detecting disrupted *HoxA1* gene. Among the three clones tested, two showed successful disruption of *HoxA1* (arrowheads). (**d**) PCR surveyor assay for detecting disrupted *CLDN2*. Among the three clones tested, two of them showed successful disruption of *CLDN2* (arrowheads). Scale bars: 100 µm.

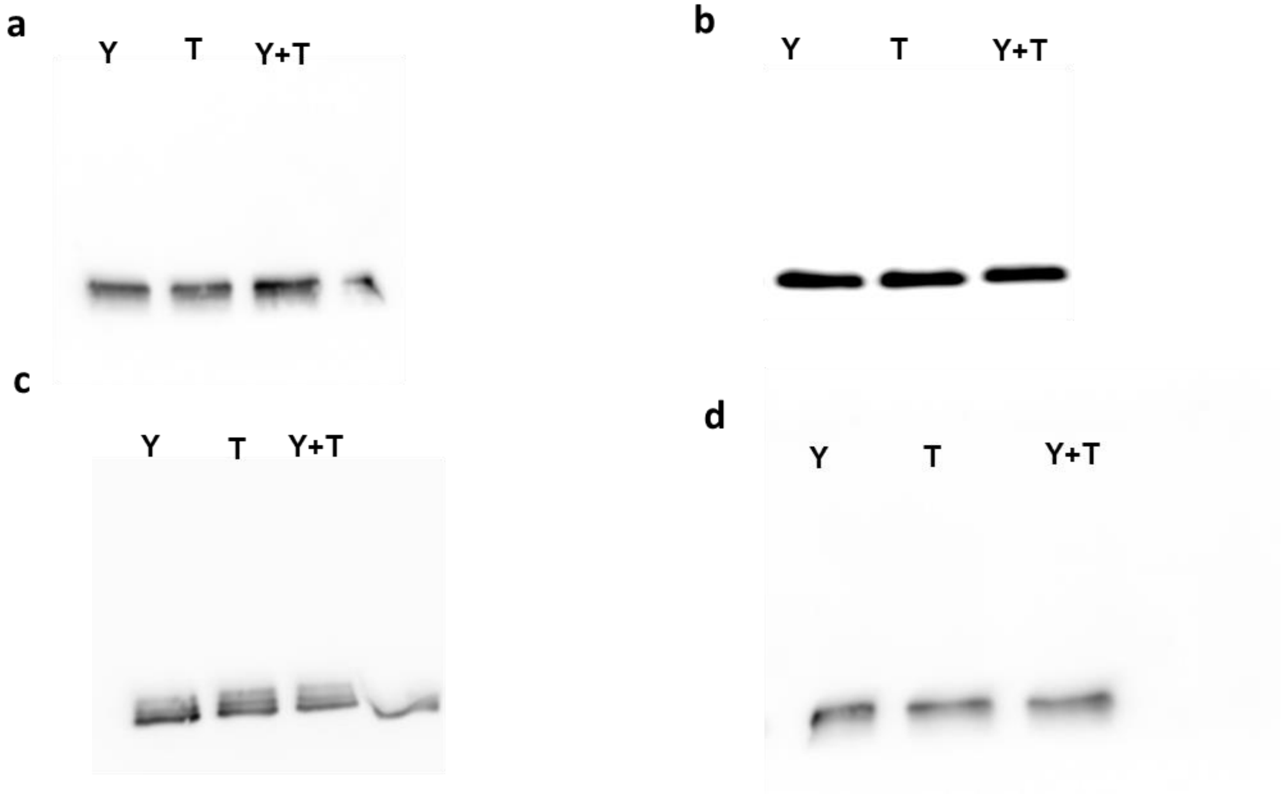


**Supplementary Figure 6**. Uncropped gel images that were shown in Supplementary Fig. S2b and c. (**a**) Uncropped gel images of beta-actin of hESCs after treatment with Y-27632 (Y), TTNPB (T) and Y-27632+TTNPB (Y+T). (**b**) Uncropped gel images of beta-actin of hiPSCs. (**c**) Uncropped gel images of Caspase-3 protein expression using Western blot from hESCs. (**d**) Uncropped gel images of Caspase-3 protein expression using Western blot from hiPSCs.

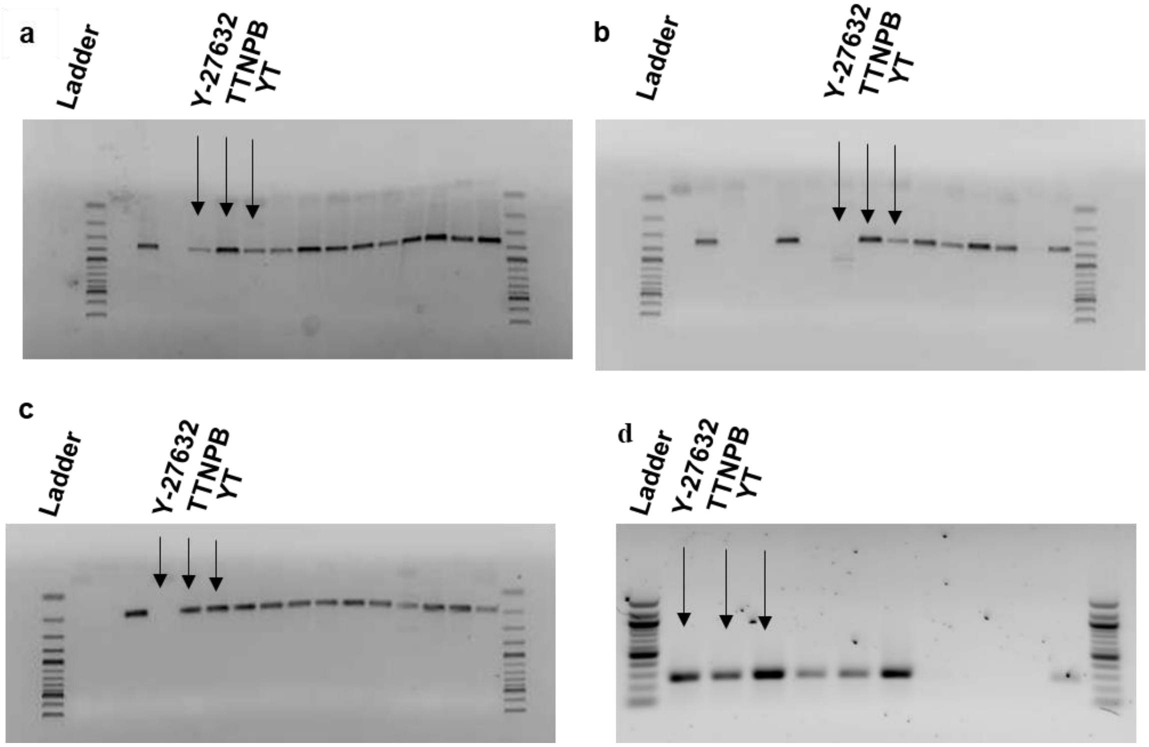


**Supplementary Figure 7**. Uncropped gel images that were shown in Supplementary Fig. S5a. (**a**) Uncropped gel images of HoxA1 after treatment with Y-27632, TTNPB and Y-27632+TTNPB. (**b**) Uncropped gel images of CLDN2 expression. (**c**) Uncropped gel images of BCL-2 expression (**d**) Uncropped gel images of GAPDH expression.

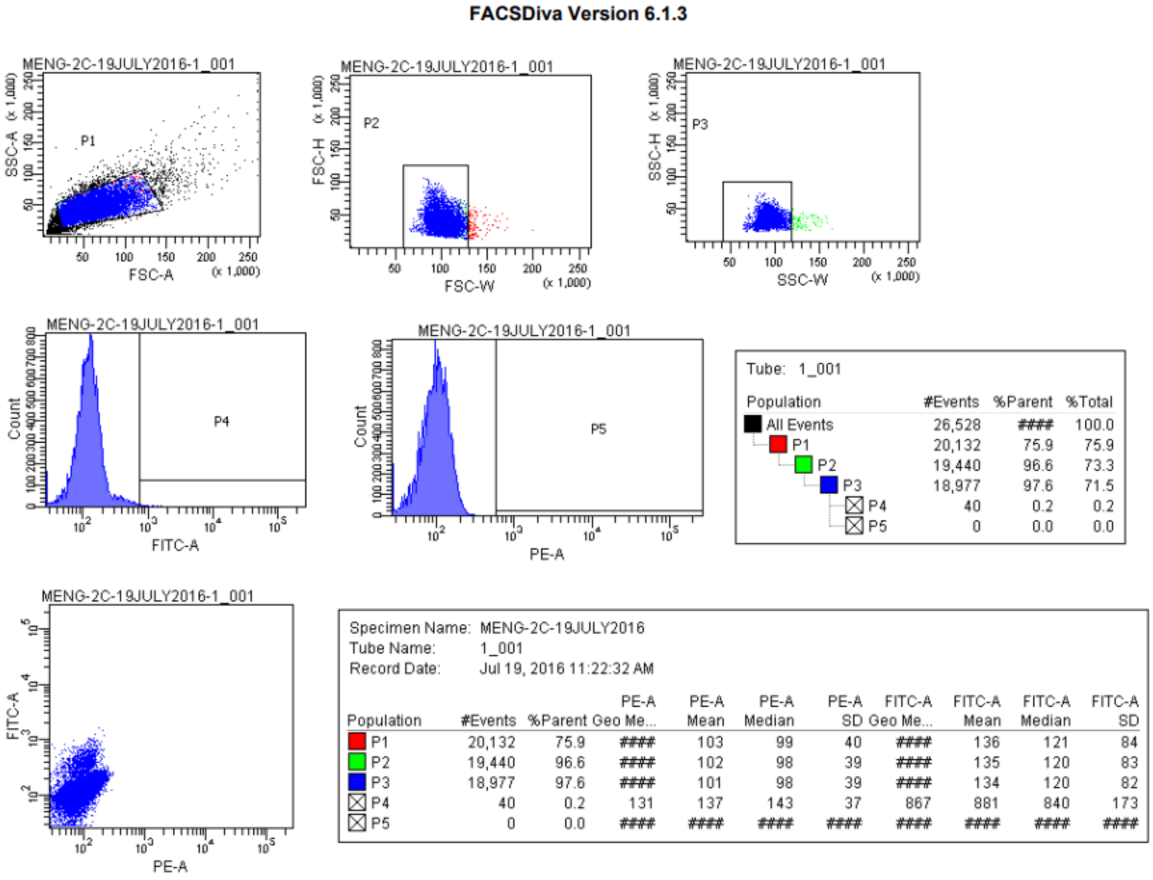


Tube: 1_001

| Population | #Events | %Parent | %Total |
|---|---|---|---|
| All Events | 26,528 | #### | 100.0 |
| P1 | 20,132 | 75.9 | 75.9 |
| P2 | 19,440 | 96.6 | 73.3 |
| P3 | 18,977 | 97.6 | 71.5 |
| P4 | 40 | 0.2 | 0.2 |
| P5 | 0 | 0.0 | 0.0 |

Specimen Name: MENG-2C-19JULY2016
Tube Name: 1_001
Record Date: Jul 19, 2016 11:22:32 AM

| Population | #Events | %Parent | PE-A Geo Me... | PE-A Mean | PE-A Median | PE-A SD | FITC-A Geo Me... | FITC-A Mean | FITC-A Median | FITC-A SD |
|---|---|---|---|---|---|---|---|---|---|---|
| P1 | 20,132 | 75.9 | #### | 103 | 99 | 40 | #### | 136 | 121 | 84 |
| P2 | 19,440 | 96.6 | #### | 102 | 98 | 39 | #### | 135 | 120 | 83 |
| P3 | 18,977 | 97.6 | #### | 101 | 98 | 39 | #### | 134 | 120 | 82 |
| P4 | 40 | 0.2 | 131 | 137 | 143 | 37 | 867 | 881 | 840 | 173 |
| P5 | 0 | 0.0 | #### | #### | #### | #### | #### | #### | #### | #### |

**Supplementary Figure 8**. Gating strategy for flow cytometry data shown in Fig. 4b. The plots are shown for unstained cells.

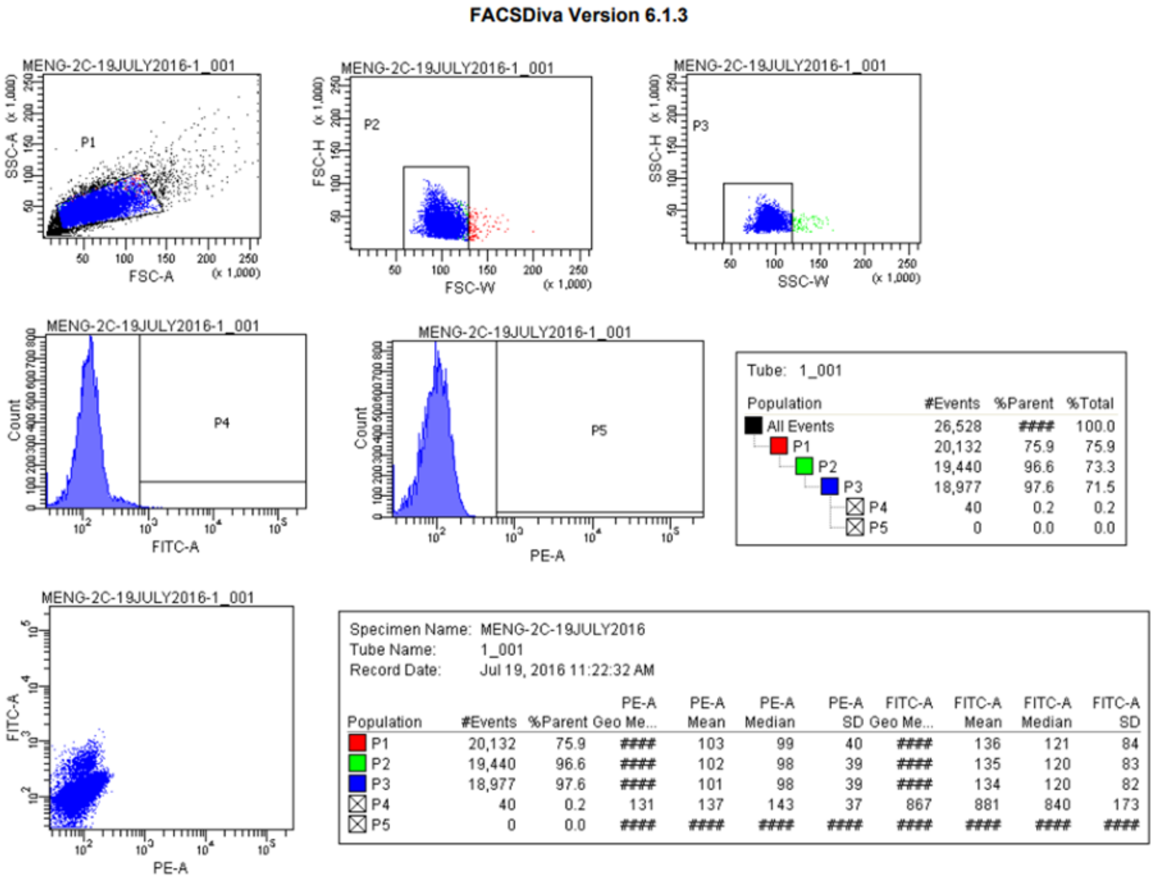


| Population | #Events | %Parent | %Total |
|---|---|---|---|
| All Events | 26,528 | #### | 100.0 |
| P1 | 20,132 | 75.9 | 75.9 |
| P2 | 19,440 | 96.6 | 73.3 |
| P3 | 18,977 | 97.6 | 71.5 |
| P4 | 40 | 0.2 | 0.2 |
| P5 | 0 | 0.0 | 0.0 |

| Population | #Events | %Parent | PE-A Geo Me... | PE-A Mean | PE-A Median | PE-A SD | FITC-A Geo Me... | FITC-A Mean | FITC-A Median | FITC-A SD |
|---|---|---|---|---|---|---|---|---|---|---|
| P1 | 20,132 | 75.9 | #### | 103 | 99 | 40 | #### | 136 | 121 | 84 |
| P2 | 19,440 | 96.6 | #### | 102 | 98 | 39 | #### | 135 | 120 | 83 |
| P3 | 18,977 | 97.6 | #### | 101 | 98 | 39 | #### | 134 | 120 | 82 |
| P4 | 40 | 0.2 | 131 | 137 | 143 | 37 | 867 | 881 | 840 | 173 |
| P5 | 0 | 0.0 | #### | #### | #### | #### | #### | #### | #### | #### |

**Supplementary Figure 9**. Gating strategy for flow cytometry data shown in Fig. 4b. The plots are shown for Oct4 negative control cells.

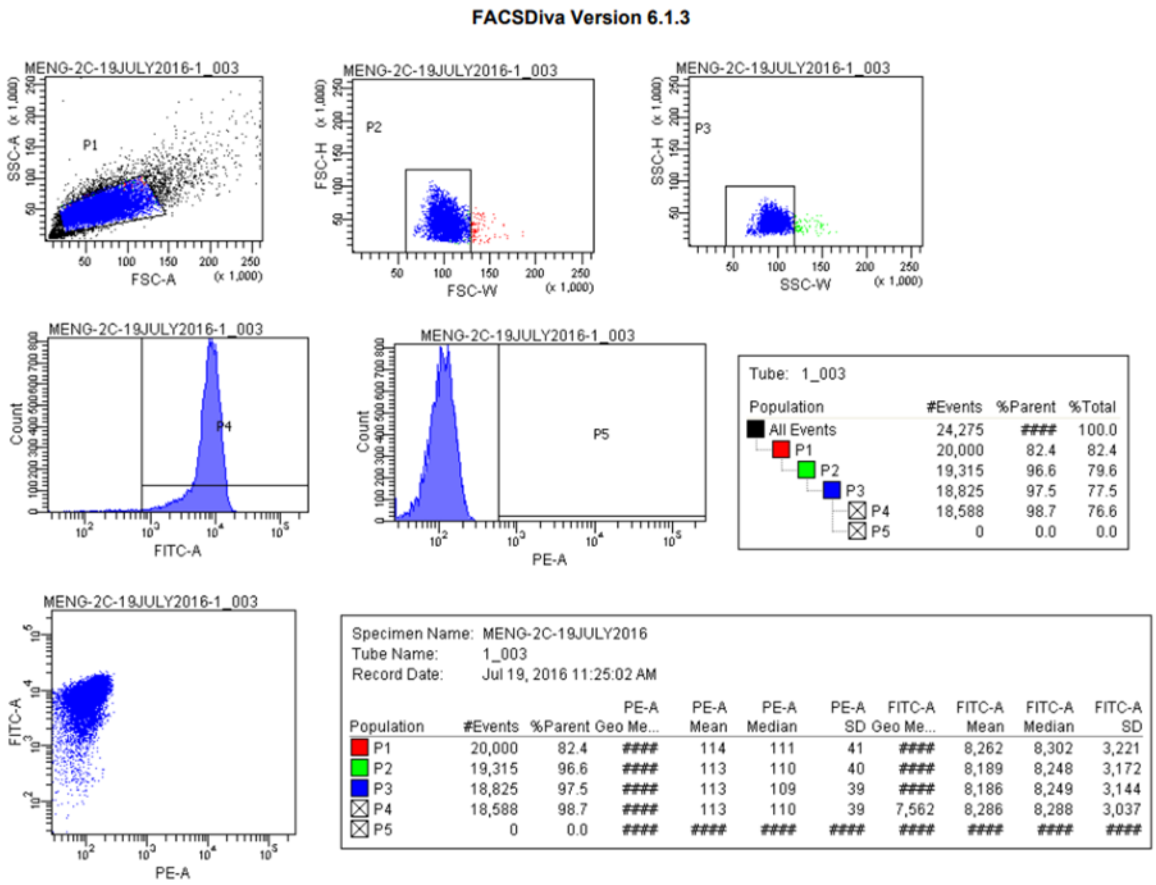


Tube: 1_003

| Population | #Events | %Parent | %Total |
|---|---|---|---|
| All Events | 24,275 | #### | 100.0 |
| P1 | 20,000 | 82.4 | 82.4 |
| P2 | 19,315 | 96.6 | 79.6 |
| P3 | 18,825 | 97.5 | 77.5 |
| P4 | 18,588 | 98.7 | 76.6 |
| P5 | 0 | 0.0 | 0.0 |

Specimen Name: MENG-2C-19JULY2016
Tube Name: 1_003
Record Date: Jul 19, 2016 11:25:02 AM

| Population | #Events | %Parent | PE-A Geo Me... | PE-A Mean | PE-A Median | PE-A SD | FITC-A Geo Me... | FITC-A Mean | FITC-A Median | FITC-A SD |
|---|---|---|---|---|---|---|---|---|---|---|
| P1 | 20,000 | 82.4 | #### | 114 | 111 | 41 | #### | 8,262 | 8,302 | 3,221 |
| P2 | 19,315 | 96.6 | #### | 113 | 110 | 40 | #### | 8,189 | 8,248 | 3,172 |
| P3 | 18,825 | 97.5 | #### | 113 | 109 | 39 | #### | 8,186 | 8,249 | 3,144 |
| P4 | 18,588 | 98.7 | #### | 113 | 110 | 39 | 7,562 | 8,286 | 8,288 | 3,037 |
| P5 | 0 | 0.0 | #### | #### | #### | #### | #### | #### | #### | #### |

**Supplementary Figure 10**. Gating strategy for flow cytometry data shown in Fig. 4b. The plots are shown for Oct4 positive cells.

**FACSDiva Version 6.1.3**

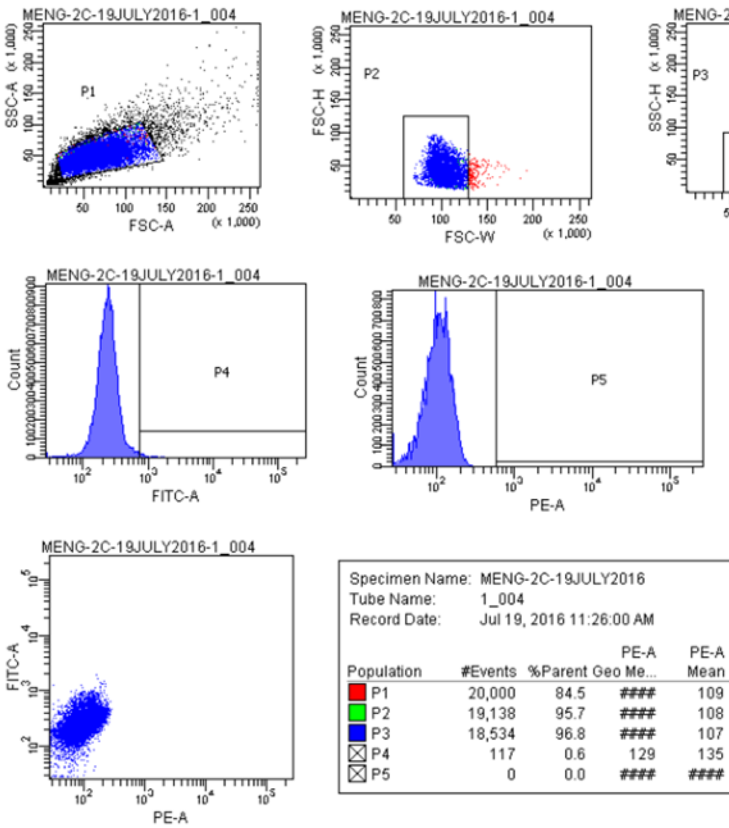


Tube: 1_004

| Population | #Events | %Parent | %Total |
|---|---|---|---|
| All Events | 23,657 | #### | 100.0 |
| P1 | 20,000 | 84.5 | 84.5 |
| P2 | 19,138 | 95.7 | 80.9 |
| P3 | 18,534 | 96.8 | 78.3 |
| P4 | 117 | 0.6 | 0.5 |
| P5 | 0 | 0.0 | 0.0 |

Specimen Name: MENG-2C-19JULY2016
Tube Name: 1_004
Record Date: Jul 19, 2016 11:26:00 AM

| Population | #Events | %Parent | PE-A Geo Me... | PE-A Mean | PE-A Median | PE-A SD | FITC-A Geo Me... | FITC-A Mean | FITC-A Median | FITC-A SD |
|---|---|---|---|---|---|---|---|---|---|---|
| P1 | 20,000 | 84.5 | #### | 109 | 105 | 40 | #### | 254 | 238 | 108 |
| P2 | 19,138 | 95.7 | #### | 108 | 104 | 39 | #### | 251 | 235 | 105 |
| P3 | 18,534 | 96.8 | #### | 107 | 104 | 39 | #### | 249 | 234 | 104 |
| P4 | 117 | 0.6 | 129 | 135 | 133 | 41 | 861 | 874 | 834 | 170 |
| P5 | 0 | 0.0 | #### | #### | #### | #### | #### | #### | #### | #### |

**Supplementary Figure 11**. Gating strategy for flow cytometry data shown in Fig. 4b. The plots are shown for Nanog negative cells.

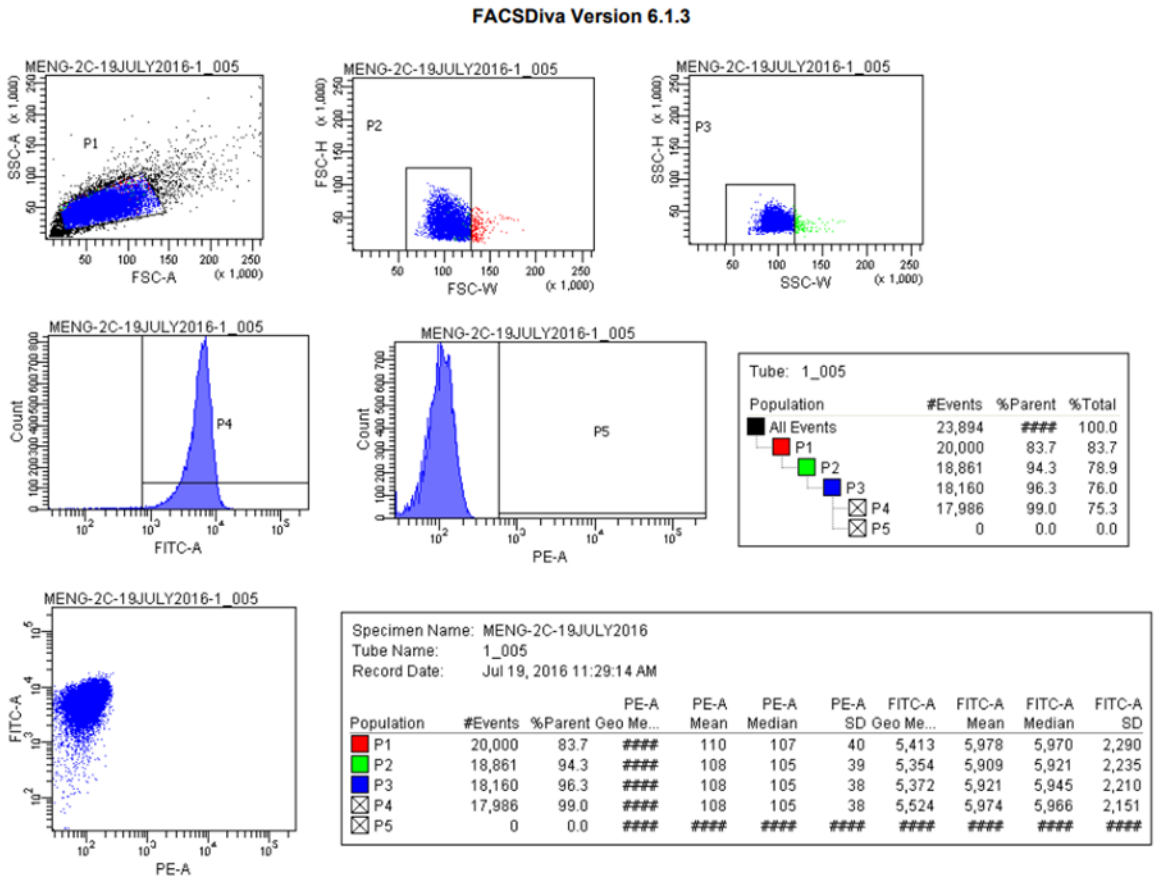


| Tube: 1_005 | | | |
|---|---|---|---|
| Population | #Events | %Parent | %Total |
| All Events | 23,894 | #### | 100.0 |
| P1 | 20,000 | 83.7 | 83.7 |
| P2 | 18,861 | 94.3 | 78.9 |
| P3 | 18,160 | 96.3 | 76.0 |
| P4 | 17,986 | 99.0 | 75.3 |
| P5 | 0 | 0.0 | 0.0 |

Specimen Name: MENG-2C-19JULY2016
Tube Name: 1_005
Record Date: Jul 19, 2016 11:29:14 AM

| Population | #Events | %Parent | PE-A Geo Me... | PE-A Mean | PE-A Median | PE-A SD | FITC-A Geo Me... | FITC-A Mean | FITC-A Median | FITC-A SD |
|---|---|---|---|---|---|---|---|---|---|---|
| P1 | 20,000 | 83.7 | #### | 110 | 107 | 40 | 5,413 | 5,978 | 5,970 | 2,290 |
| P2 | 18,861 | 94.3 | #### | 108 | 105 | 39 | 5,354 | 5,909 | 5,921 | 2,235 |
| P3 | 18,160 | 96.3 | #### | 108 | 105 | 38 | 5,372 | 5,921 | 5,945 | 2,210 |
| P4 | 17,986 | 99.0 | #### | 108 | 105 | 38 | 5,524 | 5,974 | 5,966 | 2,151 |
| P5 | 0 | 0.0 | #### | #### | #### | #### | #### | #### | #### | #### |

**Supplementary Figure 12**. Gating strategy for flow cytometry data shown in Fig. 4b. The plots are shown for Nanog positive cells.

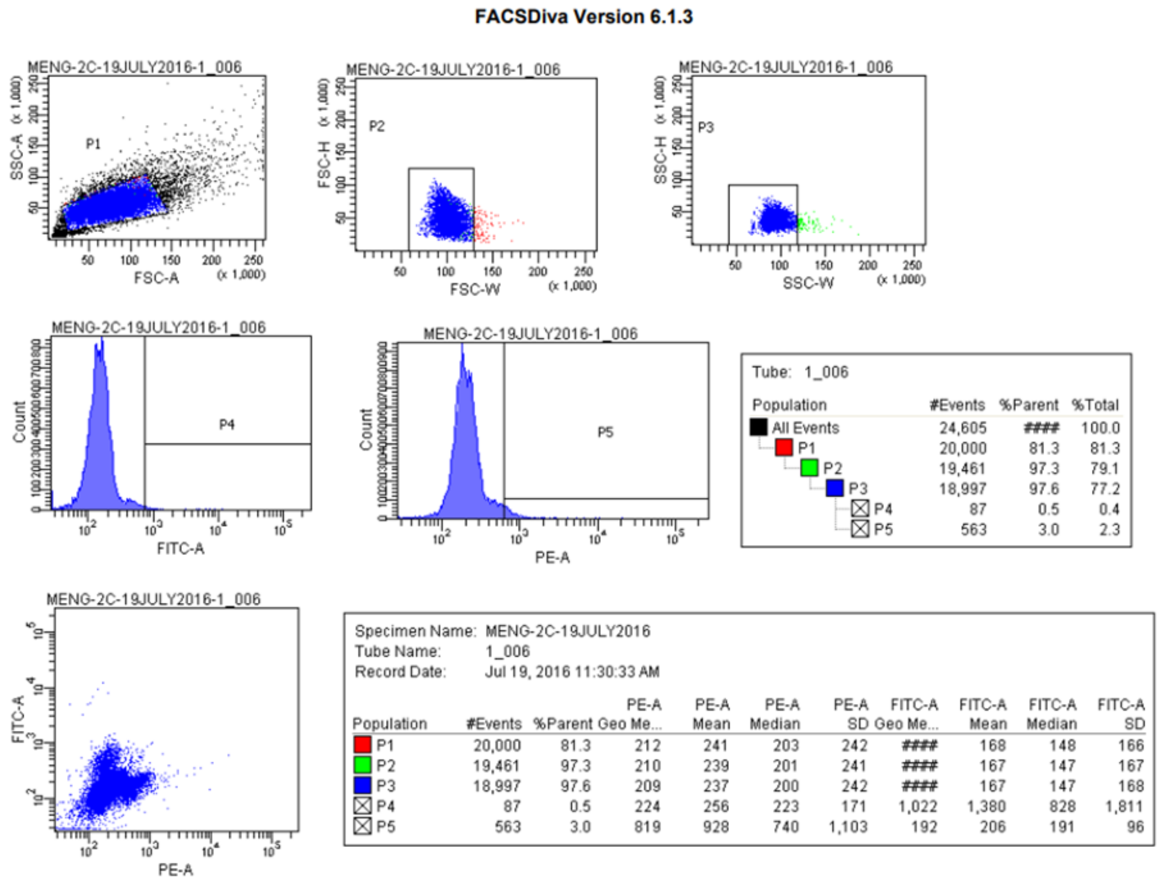


**Supplementary Figure 13**. Gating strategy for flow cytometry data shown in Fig. 4b. The plots are shown for SSEA negative cells.

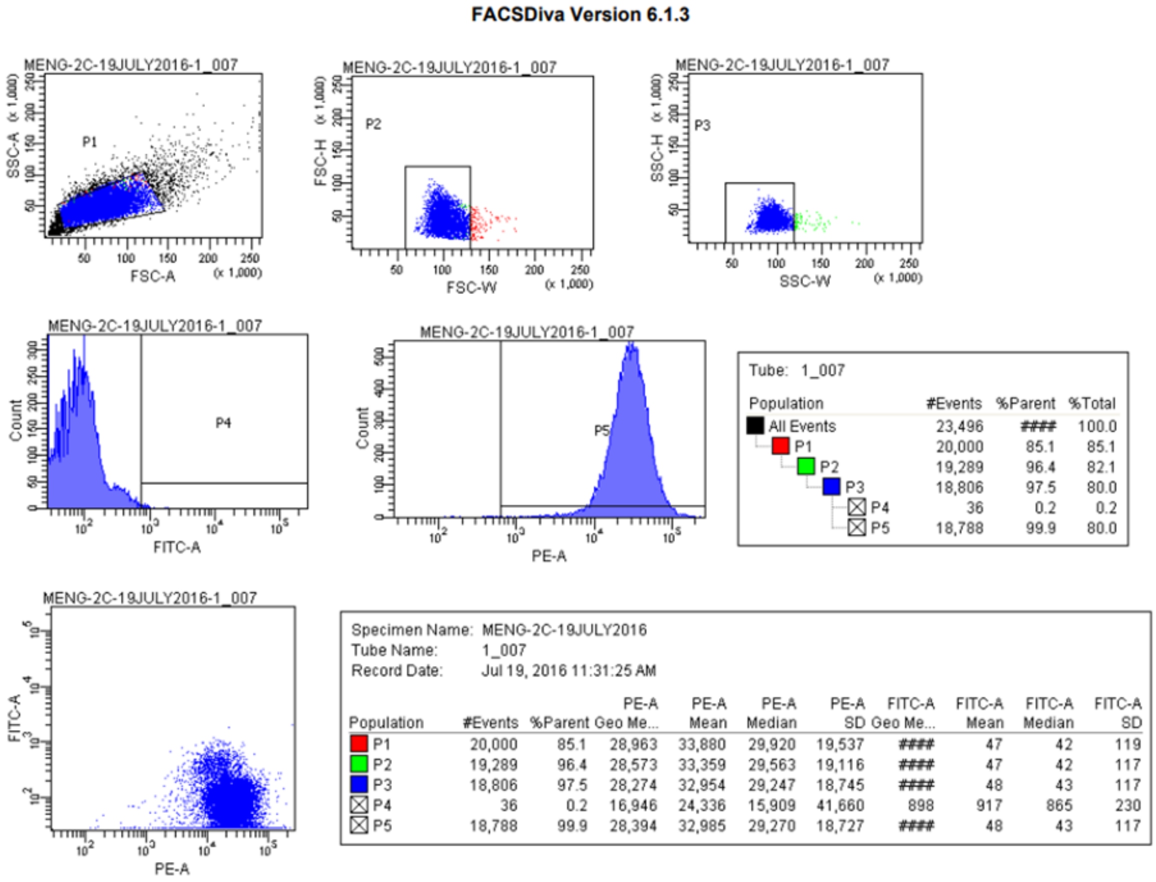


**Supplementary Figure 14**. Gating strategy for flow cytometry data shown in Fig. 4b. The plots are shown for SSEA positive cells.

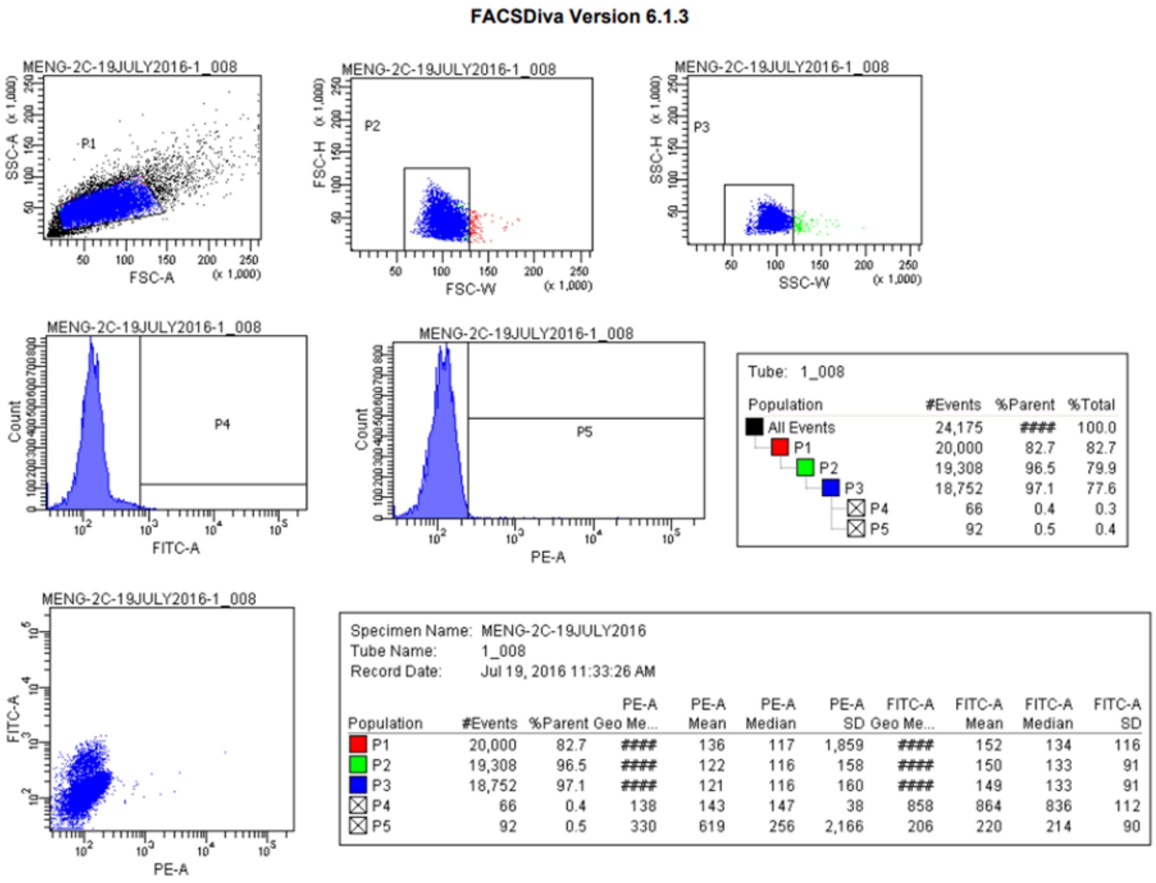


| Population | #Events | %Parent | %Total |
|---|---|---|---|
| All Events | 24,175 | #### | 100.0 |
| P1 | 20,000 | 82.7 | 82.7 |
| P2 | 19,308 | 96.5 | 79.9 |
| P3 | 18,752 | 97.1 | 77.6 |
| P4 | 66 | 0.4 | 0.3 |
| P5 | 92 | 0.5 | 0.4 |

| Population | #Events | %Parent | PE-A Geo Me... | PE-A Mean | PE-A Median | PE-A SD | FITC-A Geo Me... | FITC-A Mean | FITC-A Median | FITC-A SD |
|---|---|---|---|---|---|---|---|---|---|---|
| P1 | 20,000 | 82.7 | #### | 136 | 117 | 1,859 | #### | 152 | 134 | 116 |
| P2 | 19,308 | 96.5 | #### | 122 | 116 | 158 | #### | 150 | 133 | 91 |
| P3 | 18,752 | 97.1 | #### | 121 | 116 | 160 | #### | 149 | 133 | 91 |
| P4 | 66 | 0.4 | 138 | 143 | 147 | 38 | 858 | 864 | 836 | 112 |
| P5 | 92 | 0.5 | 330 | 619 | 256 | 2,166 | 206 | 220 | 214 | 90 |

**Supplementary Figure 15**. Gating strategy for flow cytometry data shown in Fig. 4b. The plots are shown for TRA 1-60 negative cells.

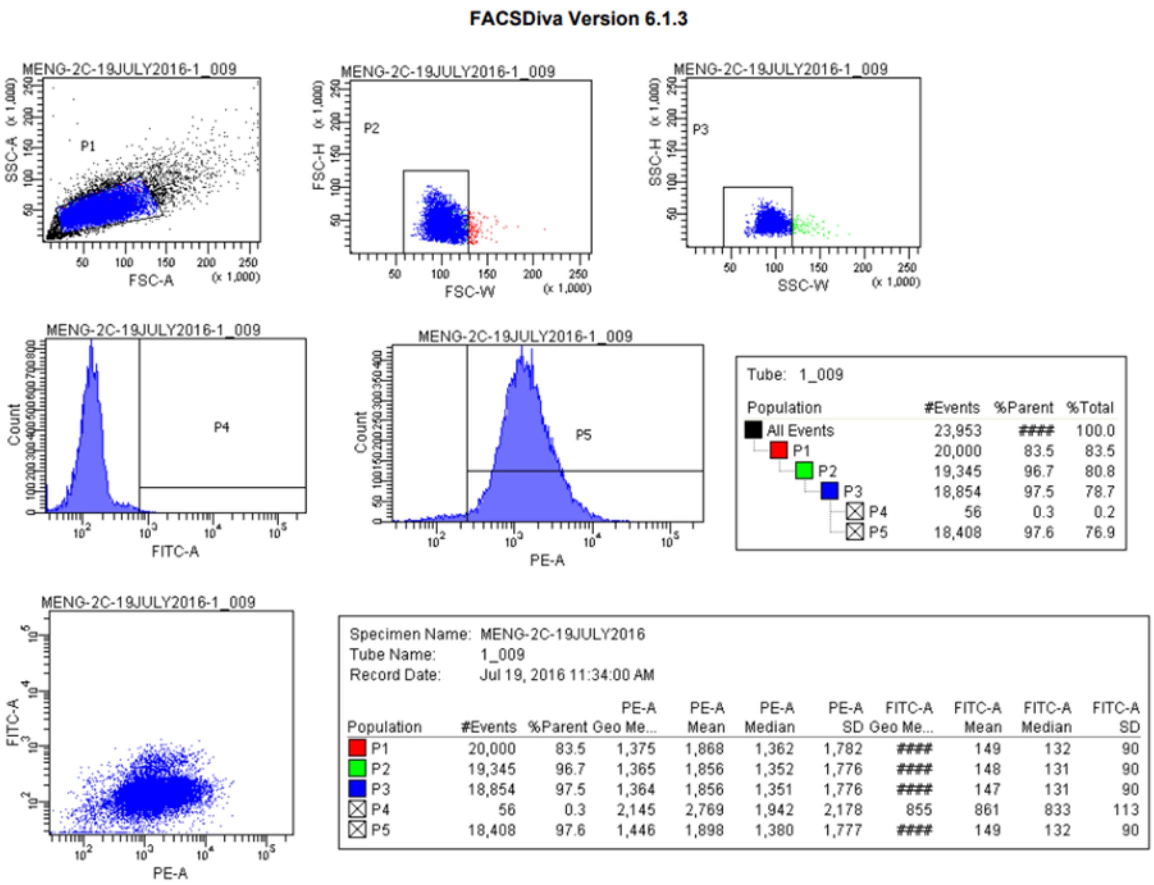


Tube: 1_009

| Population | #Events | %Parent | %Total |
|---|---|---|---|
| All Events | 23,953 | #### | 100.0 |
| P1 | 20,000 | 83.5 | 83.5 |
| P2 | 19,345 | 96.7 | 80.8 |
| P3 | 18,854 | 97.5 | 78.7 |
| P4 | 56 | 0.3 | 0.2 |
| P5 | 18,408 | 97.6 | 76.9 |

Specimen Name: MENG-2C-19JULY2016
Tube Name: 1_009
Record Date: Jul 19, 2016 11:34:00 AM

| Population | #Events | %Parent | PE-A Geo Me... | PE-A Mean | PE-A Median | PE-A SD | FITC-A Geo Me... | FITC-A Mean | FITC-A Median | FITC-A SD |
|---|---|---|---|---|---|---|---|---|---|---|
| P1 | 20,000 | 83.5 | 1,375 | 1,868 | 1,362 | 1,782 | #### | 149 | 132 | 90 |
| P2 | 19,345 | 96.7 | 1,365 | 1,856 | 1,352 | 1,776 | #### | 148 | 131 | 90 |
| P3 | 18,854 | 97.5 | 1,364 | 1,856 | 1,351 | 1,776 | #### | 147 | 131 | 90 |
| P4 | 56 | 0.3 | 2,145 | 2,769 | 1,942 | 2,178 | 855 | 861 | 833 | 113 |
| P5 | 18,408 | 97.6 | 1,446 | 1,898 | 1,380 | 1,777 | #### | 149 | 132 | 90 |

**Supplementary Figure 16**. Gating strategy for flow cytometry data shown in Fig. 4b. The plots are shown for TRA 1-60 positive cells.
