# Supplementary material for: A retinoid analogue, TTNPB, promotes clonal expansion of human pluripotent stem cells by upregulating CLDN2 and HoxA1
Source: Commun Biol. 2024 Feb 16;7:190. doi: 10.1038/s42003-024-05812-7 (PMC10873380; doi:10.1038/s42003-024-05812-7)
Supplement: Supplementary file 3 — Description of Additional Supplementary Files [file 42003_2024_5812_MOESM3_ESM.pdf]

Description of Additional Supplementary Files

File name: Supplementary Data 1
Description: The source data for the graphs and charts in the main figures**.**
