# Supplementary material for: A retinoid analogue, TTNPB, promotes clonal expansion of human pluripotent stem cells by upregulating CLDN2 and HoxA1
Source: Commun Biol. 2024 Feb 16;7:190. doi: 10.1038/s42003-024-05812-7 (PMC10873380; doi:10.1038/s42003-024-05812-7)
Supplement: Supplementary file 5 — Reporting Summary [file 42003_2024_5812_MOESM5_ESM.pdf]

nature research

Corresponding author(s): Derrick E Rancourt

Last updated by author(s): Sep 3, 2023

Reporting Summary

Nature Research wishes to improve the reproducibility of the work that we publish. This form provides structure for consistency and transparency in reporting. For further information on Nature Research policies, see our Editorial Policies and the Editorial Policy Checklist.

Statistics

For all statistical analyses, confirm that the following items are present in the figure legend, table legend, main text, or Methods section.

n/a	Confirmed	
☐	☒	The exact sample size (*n*) for each experimental group/condition, given as a discrete number and unit of measurement
☐	☒	A statement on whether measurements were taken from distinct samples or whether the same sample was measured repeatedly
☐	☒	The statistical test(s) used AND whether they are one- or two-sided<br>*Only common tests should be described solely by name; describe more complex techniques in the Methods section.*
☒	☐	A description of all covariates tested
☒	☐	A description of any assumptions or corrections, such as tests of normality and adjustment for multiple comparisons
☐	☒	A full description of the statistical parameters including central tendency (e.g. means) or other basic estimates (e.g. regression coefficient) AND variation (e.g. standard deviation) or associated estimates of uncertainty (e.g. confidence intervals)
☐	☒	For null hypothesis testing, the test statistic (e.g. *F*, *t*, *r*) with confidence intervals, effect sizes, degrees of freedom and *P* value noted<br>*Give P values as exact values whenever suitable.*
☒	☐	For Bayesian analysis, information on the choice of priors and Markov chain Monte Carlo settings
☒	☐	For hierarchical and complex designs, identification of the appropriate level for tests and full reporting of outcomes
☒	☐	Estimates of effect sizes (e.g. Cohen's *d*, Pearson's *r*), indicating how they were calculated

Our web collection on statistics for biologists contains articles on many of the points above.

Software and code

Policy information about availability of computer code

Data collection	The individual RNA-seq libraries were sequenced by NextSeq 500 (Illumina). Base-scaling and demultiplexing were done with bcl2fastq v.2.17.1.14 software (Illumina).
Data analysis	RNA-seq data was analyzed using Ingenuity Pathway Analysis software (Qiagen). Details can be found here: https://www.qiagen.com/us/products/discovery-and-translational-research/next-generation-sequencing/informatics-and-data/interpretation-content-databases/ingenuity-pathway-analysis/

For manuscripts utilizing custom algorithms or software that are central to the research but not yet described in published literature, software must be made available to editors and reviewers. We strongly encourage code deposition in a community repository (e.g. GitHub). See the Nature Research guidelines for submitting code & software for further information.

Data

Policy information about availability of data

All manuscripts must include a data availability statement. This statement should provide the following information, where applicable:

- Accession codes, unique identifiers, or web links for publicly available datasets
- A list of figures that have associated raw data
- A description of any restrictions on data availability

RNA-seq data are available in the NCBI/GEO under the accession number SUB11997390.

Field-specific reporting

Please select the one below that is the best fit for your research. If you are not sure, read the appropriate sections before making your selection.

☒ Life sciences ☐ Behavioural & social sciences ☐ Ecological, evolutionary & environmental sciences

For a reference copy of the document with all sections, see nature.com/documents/nr-reporting-summary-flat.pdf

Life sciences study design

All studies must disclose on these points even when the disclosure is negative.

Sample size	All the experiments were performed at least N=3 and shown as mean±SD.
Data exclusions	No data were excluded.
Replication	To check the reproducibility, all the experiments were performed in triplicate. We found that data are reproducible for every experiment.
Randomization	For randomization, we used two different cell lines: hESC (H9) and iPSC (4YF) to test our target drug.
Blinding	Blinding was not performed between control and target drug

Reporting for specific materials, systems and methods

We require information from authors about some types of materials, experimental systems and methods used in many studies. Here, indicate whether each material, system or method listed is relevant to your study. If you are not sure if a list item applies to your research, read the appropriate section before selecting a response.

Materials & experimental systems

n/a	Involved in the study	
☐	☒	Antibodies
☐	☒	Eukaryotic cell lines
☒	☐	Palaeontology and archaeology
☐	☒	Animals and other organisms
☒	☐	Human research participants
☒	☐	Clinical data
☒	☐	Dual use research of concern

Methods

n/a	Involved in the study	
☒	☐	ChIP-seq
☐	☒	Flow cytometry
☒	☐	MRI-based neuroimaging

Antibodies

Antibodies used	Oct4 (Milipore), Nanog (Milipore), SSEA-4 (Milipore), TRA-1-60 (Milipore), YAP (Cell Signalling), CLDN2 (Cell Signalling) and HoxA1 (SantaCruz Biotechnology)
Validation	For Oct4, Nanog, SSEA-4 and TRA-1-60, a fibroblast was used as a negative control

Eukaryotic cell lines

Policy information about cell lines

Cell line source(s)	hESC (H9) and hiPSC (4YF)
Authentication	Both cell lines were checked for pluripotency markers before starting experiment
Mycoplasma contamination	All cell lines were routinely monitored for Mycoplasma contamination
Commonly misidentified lines (See ICLAC register)	All cell lines used were identified

Animals and other organisms

Policy information about studies involving animals; ARRIVE guidelines recommended for reporting animal research

Laboratory animals	SCID mice were used for teratoma formation

Wild animals | The study did not involve wild animals

Field-collected samples | Did not involve samples collected from field

Ethics oversight | The University of Calgary Ethics Oversight Committee approved the ethics protocol for culturing hESC

Note that full information on the approval of the study protocol must also be provided in the manuscript.

Flow Cytometry

Plots

Confirm that:

- [x] The axis labels state the marker and fluorochrome used (e.g. CD4-FITC).
- [x] The axis scales are clearly visible. Include numbers along axes only for bottom left plot of group (a 'group' is an analysis of identical markers).
- [x] All plots are contour plots with outliers or pseudocolor plots.
- [x] A numerical value for number of cells or percentage (with statistics) is provided.

Methodology

Sample preparation | hESCs and hiPSCs were collected after treating with the drugs on a desired day. They were then dissociated into single cells.

Instrument | FACSAria III (BD Biosciences)

Software | FlowJo (BD Biosciences)

Cell population abundance | A minimum of 3.0e5 cells/mL was taken for flow cytometry after cell counting using an automatic cell counter

Gating strategy | Isotype was considered as negative control and used for setting up the gate

- [ ] Tick this box to confirm that a figure exemplifying the gating strategy is provided in the Supplementary Information.
